# Night work, chronotype and cortisol at awakening in female hospital employees

**DOI:** 10.1038/s41598-022-10054-w

**Published:** 2022-04-20

**Authors:** Katarzyna Burek, Sylvia Rabstein, Thomas Kantermann, Céline Vetter, Markus Rotter, Rui Wang-Sattler, Martin Lehnert, Dirk Pallapies, Karl-Heinz Jöckel, Thomas Brüning, Thomas Behrens

**Affiliations:** 1 Institute for Prevention and Occupational Medicine of the German Social Accident Insurance, Institute of the Ruhr University Bochum (IPA), Bürkle-de-la-Camp-Platz 1, 44789 Bochum, Germany; 2 Institute for Labor and Personnel (IAP), University of Applied Sciences for Economics and Management (FOM), Essen, Germany; 3 SynOpus, Bochum, Germany; 4 Department of Integrative Physiology, University of Colorado, Boulder, CO USA; 5 XIMES GmbH, Vienna, Austria; 6 Research Unit of Molecular Epidemiology, Institute of Epidemiology, Helmholtz Zentrum München - German Research Center for Environmental Health, Neuherberg, Germany; 7 Institute for Medical Informatics, Biometry and Epidemiology, University Hospital of Essen, University Duisburg-Essen, Essen, Germany

**Keywords:** Epidemiology, Endocrinology, Health occupations

## Abstract

To examine the effect of night shift on salivary cortisol at awakening (C1), 30 min later (C2), and on the cortisol awakening response (CAR, the difference between C2 and C1). We compared shift and non-shift workers with a focus on the impact of worker chronotype. Our study included 66 shift-working females (mean age = 37.3 years, SD = 10.2) and 21 non-shift working females (mean age = 47.0 years, SD = 8.9). The shift workers collected their saliva samples at C1 and C2 on each two consecutive day shifts and night shifts. Non-shift workers collected their samples on two consecutive day shifts. We applied linear mixed-effects models (LMM) to determine the effect of night shift on CAR and log-transformed C1 and C2 levels. LMMs were stratified by chronotype group. Compared to non-shift workers, shift workers before day shifts (i.e. after night sleep) showed lower cortisol at C1 (exp $(\widehat{\beta })$=0.58, 95% CI 0.42, 0.81) but not at C2. In shift workers, the CARs after night shifts (i.e. after day sleep) were lower compared to CARs before day shifts ($\widehat{\beta }$= − 11.07, 95% CI − 15.64, − 6.50). This effect was most pronounced in early chronotypes (early: $\widehat{\beta }$= − 16.61, 95% CI − 27.87, − 5.35; intermediate: $\widehat{\beta }$= − 11.82, 95% CI − 18.35, − 5.29; late: $\widehat{\beta }$= − 6.27, 95% CI − 14.28, 1.74). Chronotype did not modify the association between night shift and CAR. In our population of shift workers, there was a mismatch between time of waking up and their natural cortisol peak at waking up (CAR) both during day and night shift duties.

## Introduction

Working in shifts and especially at night impedes a regular sleep–wake timing, which, in effect, may compromise the process of entrainment of the circadian clock^1,2^. During night shifts individuals work, eat, drink and are exposed to light at times they usually sleep. In addition, sleep often is shifted into the day. These alterations may adversely affect the balance of hormones (e.g. cortisol) and physiological processes for restorative sleep and the body’s homeostasis, which in turn may increase the risk of insomnia, accidents, metabolic and cardiovascular problems, depression, and cancer^1,3,4^. Those working frequently at night may also risk other people’s health due to impaired performance (e.g. compromising patient safety in hospitals)^5^. Obviously, shift work cannot be abolished completely, since many sectors (such as transportation, healthcare, first responders) require 24/7 operations. Therefore, effective solutions need to consider how shift work impacts on physiological systems that help to maintain the body’s state of arousal and alertness^4,6^.

The secretion of the glucocorticoid cortisol is the result of rhythmic activity of the hypothalamic–pituitary–adrenal axis, which is controlled by the master clock in the suprachiasmatic nuclei^7^. In humans, cortisol naturally shows circadian rhythmicity with an early-morning peak after night sleep, declining levels throughout the day, a quiescent period of minimal secretory activity early at night, and, again, increasing levels in anticipation of waking up^8^. The sharp increase in cortisol over the first 30–45 min after morning awakening is described as the cortisol awakening response (CAR)^9,10^. An attenuated CAR has been related to a range of physiological (e.g. cardiovascular, BMI/obesity, autoimmune, allergic) and psychological factors (e.g. chronic stress, fatigue, burnout, exhaustion)^11–13^. In addition to the strong circadian component in cortisol secretion, complexity arises from the finding that individuals can differ in the phase of entrainment of their circadian clock and in their sleep timing. The individual variation in the phase of entrainment has been referred to as chronotype differences^14^, which consequently means that the local time point at which the CAR occurs varies between individuals.

A decreased CAR after night shifts (i.e. after day sleep) has been found in field studies on diverse shift-work populations of, for example, manufacturing^15^ and offshore workers^16^, nurses^17,18^ and police workers^19,20^. Kudielka et al.^15^ showed a decreased CAR after night shifts in workers that changed from permanent day shifts to a fast rotating schedule. In another study, a lower CAR was shown in night workers compared to workers on early and late shifts^19,20^. Shifts of the cortisol rhythm are not expected within the first two days of night shift^18,21,22^, because of the slow adaptation pace of the circadian system^23^. Niu et al.^18^ found differences in the CAR between night and day workers not before their third workday. Little is known whether chronotype modulates the effect of night shift on CAR. Dockray and Steptoe^24^ reported no chronotype differences in cortisol among non-shift working women, which contrasts findings by Kudielka et al.^25^ and Petrowski et al.^26^, showing higher cortisol levels after waking up from night sleep in early chronotypes. Minelli et al.^27^ in nurses working a fast-rotating shift schedule found that total cortisol output during night shifts correlated with chronotype.

In the present study, we examined the effect of waking up from day sleep after night shifts on CAR (primary outcome) and salivary cortisol at wake time (C1) and 30 min after (C2) in female hospital employees working both day and night shifts, taking chronotype into account. Chronotype was assessed using the Munich ChronoType Questionnaire for shift workers (MCTQ_shift_)^28^. We were able to compare CAR, C1, and C2 levels between day and night shifts in the same individuals, to add to the understanding of cortisol differences in shift workers with different chronotypes. In addition, we analysed differences in CAR and salivary cortisol levels between day shifts of two groups of workers: those working both day and night shifts, and those with day schedules only.

## Results

**Figure 1:**
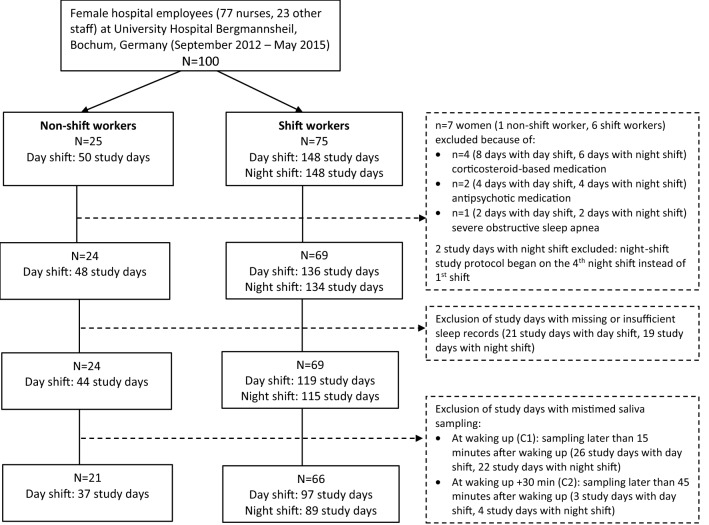
Flow chart of study population and exclusions for the final analysis dataset.

Figure 1 illustrates the number of study days available for the two study groups and the construction of the final dataset for analysis. Among the 100 women (25 non-shift workers, 75 shift workers), we excluded four women with intake of asthma medication including glucocorticoids, two with antipsychotic medication, and one with severe obstructive sleep apnea. Two night-shift study days were discarded because one woman started the study protocol on the fourth night shift instead of the first (non-adherence to study protocol). We used home polysomnography recorded time of awakening and self-reported sampling times of saliva samples at waking up (C1) and 30 min after waking up (C2) to calculate delays between awakening and both C1 and C2. We excluded study days with missing or insufficient sleep records (21 study days with day shift, 19 study days with night shift). Study days with sampling delays exceeding an accuracy margin of ± 15 min for C1 and delays exceeding 45 min for C2 according to polysomnography were excluded (29 study days with day shift, 26 study days with night shift). Thus, 21 women (eight nurses, ten medical lab assistants, three in administration) in the non-shift workers group (37 study days with day shift) and 66 women (59 nurses and seven medical lab assistants) in the shift-work group (97 study days with day shift, 89 study days with night shift) were included into the final analysis. In non-shift workers, mean saliva sampling time for C1 was 5:23 h ± (SD) 0:36 and for C2 5:53 h ± (SD) 0:35. During day shifts in shift workers, mean saliva sampling time for C1 was 4:43 h ± (SD) 0:20 and for C2 5:14 h ± (SD) 0:19. During night shifts, mean saliva sampling time for C1 was 14:38 h ± (SD) 1:30 and for C2 15:12 h ± (SD) 1:30.

Age, chronotype and menopausal status differed between the study groups (Table 1). Shift workers were younger and had later chronotypes than non-shift workers. Mean chronotype ± SD was 4:17 h ± 1:16 for shift workers and 3:15 h ± 1:18 for non-shift workers, respectively (*p* = 0.002). Less than half of the participants (n = 29, 43.9% of shift workers; and n = 10, 47.6% of non-shift workers) were overweight/obese (BMI ≥ 25 kg/m^2^). Of those being overweight/obese, 43.6% had mild or moderate OSA (n = 12, 18.2% shift workers; and n = 5, 23.8% of non-shift workers). The chronotypes of the shift and non-shift workers were: 19.7% (n = 13) and 42.9% (n = 9) with early, 48.5% (n = 32) and 42.9% (n = 9) with intermediate, 31.8% (n = 21) and 4.8% (n = 1) with late chronotype, respectively. In non-shift workers, the early chronotype group tended being more overweight (mean BMI ± SD for early 29.1 ± 6.6, and intermediate 24.4 ± 3.4). Sleep medication was only reported for study days with night shifts (n = 5 study days, 5.6%). Alcohol consumption prior to sleep was only reported on study days with day shifts (shift workers: n = 10, 10.3%; non-shift workers: n = 9, 24.3%).

**Table 1:** Characteristics of study participants (N = 87) by study group and stratified by chronotype category.

	Non-shift workers				Shift workers				*p*-value^a^
	All^b^	Early	Intermediate	Late	All	Early	Intermediate	Late	
N (%)	21 (100%)	9 (42.9%)	9 (42.9%)	1 (4.8%)	66 (100%)	13 (19.7%)	32 (48.5%)	21 (31.8%)	–
Study days	37 (100%)	16 (43.2%)	15 (40.5%)	2 (5.4%)	186 (100%)	41 (22.0%)	95 (51.1%)	50 (26.9%)	–
Age (years)	47.0 (8.9)	45.6 (8.1)	50.9 (7.2)	–	37.3 (10.2)	45.5 (5.2)	38.0 (10.5)	31.2 (8.3)	0.0002
Chronotype^b^ (MCTQ_shift_) [hh:mm]	3:15 (1:18)	2:22 (1:13)	3:57 (0:23)	–	4:17 (1:16)	2:39 (0:41)	4:00 (0:23)	5:43 (0:47)	0.0020
**Body-mass-index (kg/m**^**2**^**)**	26.9 (6.1)	29.1 (6.6)	24.4 (3.4)	–	26.1 (5.1)	25.2 (4.22)	26.8 (5.3)	25.6 (5.3)	0.5576
Normal weight (< 25 kg/m^2^)	11 (52.4%)	3 (33.3%)	6 (66.7%)		37 (56.1%)	7 (53.8%)	17 (53.1%)	13 (61.9%)	0.7914
Overweight (25–29.9 kg/m^2^)	4 (19.0%)	2 (22.2%)	2 (22.2%)		15 (22.7%)	4 (30.8%)	7 (21.9%)	4 (19.0%)	
Obesity (≥ 30 kg/m^2^)	6 (28.6%)	4 (44.4%)	1 (11.1%)		14 (21.2%)	2 (15.4%)	8 (25.0%)	4 (19.0%)	
**Obstructive sleep apnea**									
None	12 (57.1%)	6 (66.7%)	3 (33.3%)	–	47 (71.2%)	10 (76.9%)	23 (71.9%)	14 (66.7%)	0.2119
Mild	6 (28.6%)	2 (22.2%)	4 (44.4%)		16 (24.2%)	3 (23.1%)	8 (25.0%)	5 (23.8%)	
Moderate	3 (14.3%)	1 (11.1%)	2 (22.2%)		3 (4.6%)	0	1 (3.1%)	2 (9.5%)	
**Menopausal status**									
Premenopausal	9 (42.9%)	5 (55.6%)	3 (33.3%)	–	58 (87.9%)	11 (84.6%)	27 (84.4%)	20 (95.2%)	< 0.0001
Postmenopausal^c^	6 (28.6%)	1 (11.1%)	3 (33.3%)		7 (10.6%)	1 (7.7%)	5 (15.6%)	1 (4.8%)	
Surgical/other amenorrhea	6 (28.6%)	3 (33.3)	3 (33.3%)		1 (1.5%)	1 (7.7%)	0	0	
**Smoking status**									
Never	10 (47.6%)	5 (55.6%)	4 (44.4%)	–	32 (48.5%)	8 (61.5%)	15 (46.9%)	9 (42.9%)	0.1445
Former	7 (33.3%)	3 (33.3%)	3 (33.3%)		10 (15.1%)	3 (23.1%)	4 (12.5%)	3 (14.3%)	
Current	4 (19.1%)	1 (11.1%)	2 (22.2%)		24 (36.4%)	2 (15.4%)	13 (40.6%)	9 (42.9%)	

N (%) for categorical variables or mean (SD) for continuous variables.

*MCTQ*_*shift*_, Munich ChronoType Questionnaire for shift workers.

^a^*p*-values for Fisher’s exact test for categorical variables or for two-sample t-test testing differences in means between non-shift and shift workers.

^b^Two non-shift workers with missing chronotype.

^c^Self-report of natural menopause.

### Sleep and cortisol

Table 2 summarizes sleep characteristics and awakening salivary-cortisol levels with respect to study day, group and shift. Bedtime and time of waking up varied with both shift type and start time, but not between the two study days within each group. Day sleep duration after night shifts differed between study days 1 and 2. No such difference was observed for night sleep duration after day shifts, neither in non-shift workers nor in shift workers. With respect to chronotype, there were no differences in bedtime, time of waking up and sleep duration in each of the three groups (Supplementary Fig. S2). In shift workers, differences in CAR were observed between day and night shift. Negative CARs occurred more frequently on study days with night shifts (Table 2). In night shifts, negative CARs occurred more frequently in early chronotypes (early 33.3%, intermediate 19.6%, and late 12.0%).

**Table 2:** Sleep characteristics and awakening salivary cortisol measures by study day, group and shift.

	Non-shift workers (N = 21)	Shift workers (N = 66)		*p*-value^a^	*p*-value^b^
	Day shift	Day shift	Night shift		
**Study days, n (%)**					
Total	37 (100%)	97 (100%)	89 (100%)	n/a	n/a
Day 1	19 (51.4%)	46 (47.4%)	48 (53.9%)		
Day 2	18 (48.6%)	51 (52.6%)	41 (46.1%)		
**Season of sampling, n (%)**					
Spring	6 (16.2%)	21 (21.7%)	33 (37.1%)	0.4312	0.0095
Summer	13 (35.1%)	24 (24.7%)	21 (23.6%)		
Fall	4 (10.8%)	19 (19.6%)	22 (24.7%)		
Winter	14 (37.8%)	33 (34.0%)	13 (14.6%)		
*Sleep parameters*	*Mean (SD)*	*Mean (SD)*	*Mean (SD)*		
**Bedtime (clock time)**					
Day 1	22:33 (0:55)	22:11 (0:42)	8:10 (1:06)	0.0068	< 0.0001
Day 2	22:37 (1:13)	22:06 (0:46)	8:08 (1:01)		
*p*-value^c^	0.9963	0.9745	0.994		
**Waking up (clock time)**					
Day 1	5:22 (0:37)	4:44 (0:19)	14:31 (1:36)	< 0.0001	< 0.0001
Day 2	5:23 (0:34)	4:43 (0:22)	14:47 (1:21)		
*p*-value^c^	0.9958	0.9994	0.6347		
**Sleep duration (hh:mm)**					
Day 1	5:49 (1:02)	5:43 (0:53)	5:29 (1:26)	0.2679	0.7499
Day 2	6:03 (1:14)	5:45 (0:49)	6:06 (1:01)		
*p*-value^c^	0.8598	0.9988	0.034		
*CAR*^*†*^* (nmol/l)*					
**Negative, n (%)**					
Day 1	1 (2.7%)	1 (1.0%)	10 (11.2%)	n/a	n/a
Day 2	1 (2.7%)	3 (3.1%)	8 (9.0%)		
**Mean (SD)**					
Day 1	16.36 (16.46)	21.04 (16.21)	8.41 (10.75)	0.3226	< 0.0001
Day 2	17.30 (15.63)	18.47 (12.96)	8.82 (12.72)		
*p*-value^c^	0.9976	0.7758	0.9987		
*Salivary cortisol*	*GM (GSD)*	*GM (GSD)*	*GM (GSD)*		
**C1: at waking up (nmol/l)**					
Day 1	16.00 (1.70)	9.20 (1.99)	11.15 (1.79)	< 0.0001	0.1973
Day 2	20.89 (1.62)	12.44 (2.02)	13.13 (1.85)		
*p*-value^c^	0.5901	0.1012	0.6348		
**C2: at waking up + 30 min (nmol/l)**					
Day 1	32.07 (1.41)	27.32 (2.05)	18.01 (1.89)	0.1247	0.0006
Day 2	36.37 (1.58)	28.98 (1.99)	22.03 (1.74)		
*p*-value^c^	0.9308	0.9686	0.4749		

*SD* standard deviation; *GM* geometric mean; *GSD* geometric standard deviation; *CAR* cortisol awakening response; *n/a* not available.

Linear mixed models *p*-values were multiplicity adjusted.

^a^*p*-values for Chi-square test for categorical variables or for linear mixed models for continuous variables testing differences in means or geometric means between day shifts of non-shift and shift workers.

^b^*p*-values for Chi-square test for categorical variables or for linear mixed models for continuous variables testing differences in means or geometric means between day and night shift within shift workers.

^c^Linear mixed models *p*-value testing differences in means or geometric means between study day 1 and 2.

### Effect of different day shift conditions

**Figure 2:**
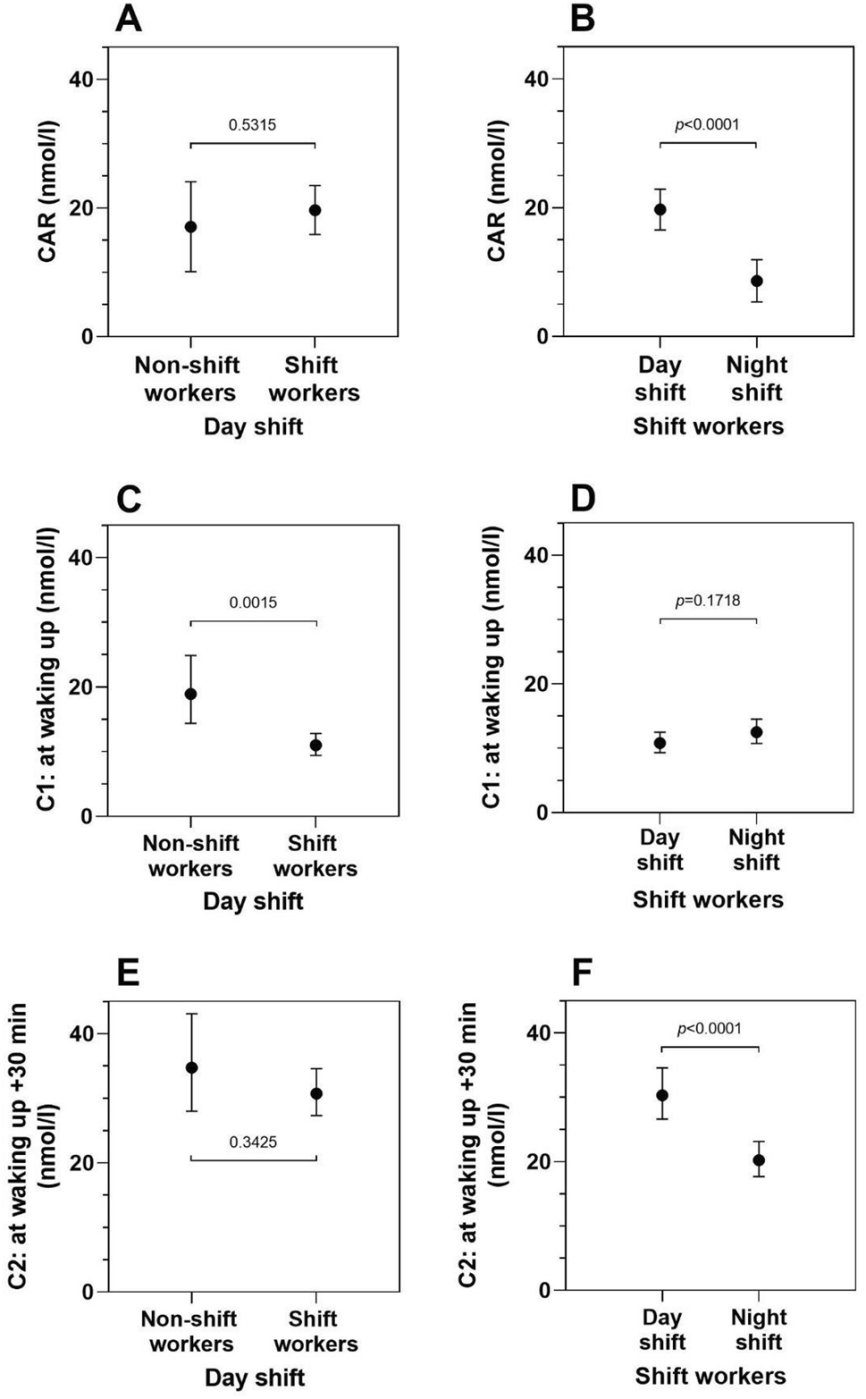
Adjusted least-squares means (LS-means) and 95% confidence intervals of salivary cortisol levels in non-shift and shift workers on day shifts (**A**, **C**, **E**) and shift workers only on day and night shifts (**B**, **D**, **F**) with CAR, C1, and C2. LS-means are derived from linear mixed-effects models as shown in Table 3.

Table 3 summarizes the associations between study group and the three cortisol measures (C1, C2, CAR). Figure 2A,C,E display these findings. Shift workers after night sleep showed lower cortisol levels at C1 compared to non-shift workers after night sleep (exp $(\widehat{\beta })$=0.58, 95% CI 0.42, 0.81). Cortisol at C1 was higher on the 2nd study day compared to 1st study day (exp $(\widehat{\beta })$=1.35, 95% CI 1.13, 1.60) (Table 3, Fig. 2C). Stratifying by either BMI or season yielded comparable results (Supplementary Tables S1 and S2).

**Table 3:** Associations (effect estimates and 95% confidence intervals) of day shift and of night shift with CAR, log-transformed salivary cortisol at waking up (C1) and 30 min after waking up (C2).

	Non-shift and shift workers on day shifts (n = 134 study days)		Shift workers on day and night shifts (n = 186 study days)	
**CAR**^**ac**^	$\widehat{\beta }$(95% CI)	*p*-value	$\widehat{\beta }$(95% CI)	*p*-value
*Fixed effects*				
Intercept	18.07 (− 8.28, 44.41)	0.1757	12.58 (− 4.26, 29.41)	0.1417
**Day shift**				
Non-shift workers	0			
Shift workers	2.58 (− 5.65, 10.82)	0.5336	n.a.	n.a.
**Shift type**				
Day shift			0	
Night shift	n.a.	n.a.	− 11.07 (− 15.64, − 6.50)	< 0.0001
**Study day**				
Day 1	0		0	
Day 2	− 1.66 (− 5.40, 2.07)	0.3766	− 1.17 (− 4.02, 1.67)	0.4154
* Random effects*	*Variance component (SE)*		*Variance component (SE)*	
Intercept	128.9 (32.6)		n.a.	
Shift type	n.a.		88.86 (20.09)	
Residual	100.9 (18.8)		85.43 (13.29)	
**C1: at waking up**^**bc**^	exp ($\widehat{\beta }$)(95% CI)	*p*-value	exp ($\widehat{\beta }$)(95% CI)	*p*-value
*Fixed effects*				
Intercept	41.45 (15.9, 108.1)	< 0.0001	32.73 (15.08, 71.03)	< 0.0001
**Day shift**				
Non-shift workers	1			
Shift workers	0.58 (0.42, 0.81)	0.0014	n.a.	n.a.
**Shift type**				
Day shift			1	
Night shift	n.a.	n.a.	1.16 (0.94, 1.43)	0.1727
**Study day**				
Day 1	1		1	
Day 2	1.35 (1.13, 1.60)	0.0012	1.25 (1.09, 1.44)	0.0021
* Random effects*	*Variance component (SE)*		*Variance component (SE)*	
Intercept	0.1664 (0.05)		n.a.	
Shift type	n.a.		0.1731 (0.05)	
Residual	0.2321 (0.04)		0.2119 (0.03)	
**C2: at waking up + 30 min**^**bc**^	exp ($\widehat{\beta }$)(95% CI)	*p*-value	exp ($\widehat{\beta }$)(95% CI)	*p*-value
*Fixed effects*				
Intercept	41.17 (17.91, 94.64)	< 0.0001	39.11 (19.49, 78.47)	< 0.0001
**Day shift**				
Non-shift workers	1			
Shift workers	0.88 (0.69, 1.14)	0.3422	n.a.	n.a.
**Shift type**				
Day shift			1	
Night shift	n.a.	n.a.	0.67 (0.55, 0.80)	< 0.0001
**Study day**				
Day 1	1		1	
Day 2	1.07 (0.97, 1.19)	0.1764	1.12 (0.99, 1.26)	0.0620
* Random effects*	*Variance component (SE)*		*Variance component (SE)*	
Intercept	0.1317 (0.03)		n.a.	
Shift type	n.a.		0.1478 (0.04)	
Residual	0.0781 (0.01)		0.1424 (0.02)	

*CAR* cortisol awakening response; *CI* confidence interval; *SE* standard error; *n.a.* not applicable.

^a^Results of linear mixed models for CAR as outcome, reported as effect estimate ($\widehat{\beta }$) with 95% CI.

^b^Results of linear mixed models for log(C1) or log(C2) as outcomes, reported as back-transformed effect estimates (exp $(\widehat{\beta })$) and 95% CI.

^c^Adjusted for age, and chronotype (MCTQ_shift_, clock times).

### Effect of night shift in shift workers

Night shifts were negatively associated with CAR ($\widehat{\beta }$= − 11.07, 95% CI − 15.64, − 6.50) (Table 3, Fig. 2B). Night shifts were associated with slightly higher cortisol at C1 (exp $(\widehat{\beta })$=1.16, 95% CI 0.94, 1.43) and lower cortisol at C2 (exp $(\widehat{\beta })$=0.67, 95% CI 0.55, 0.80) (Table 3, Fig. 2D,F). In spring/summer the negative association between night shift and CAR was attenuated ($\widehat{\beta }$= − 7.78, 95% CI − 13.63, − 1.92) (Supplementary Table S2). Excluding days with negative CAR, diminished the negative association between night shift and CAR and the association with cortisol at C1 disappeared. In contrast, C2 levels remained unchanged (Supplementary Table S3). Excluding days of participants who reported sleep medication or alcohol consumption prior their sleep, did not alter the results (data not shown).

### Effect of night shift in shift workers by chronotype

Negative associations of night shift with CAR were most pronounced in early chronotypes ($\widehat{\beta }$= − 16.61, 95% CI − 27.87, − 5.35) (Table 4). For early chronotypes we observed a negative association between the 2nd compared to the 1st study day and CAR ($\widehat{\beta }$= − 7.47, 95% CI − 14.79, − 0.15) and higher cortisol at C1 (exp $(\widehat{\beta })$=1.63, 95% CI 1.10, 2.42). In the sensitivity analysis excluding study days with negative CARs, the magnitude of the association between night shift and CAR was similar in early and intermediate chronotypes (Supplementary Table S4).

**Table 4:** Associations (effect estimates and 95% confidence intervals) of night shift with CAR, log-transformed salivary cortisol at waking up (C1) and 30 min after waking up (C2) stratified by chronotype group.

	Early chronotype (n = 41)		Intermediate chronotype (n = 95)		Late chronotype (n = 50)	
**CAR**^**a**^	$\widehat{\beta }$(95% CI)	*p*-value	$\widehat{\beta }$(95% CI)	*p*-value	$\widehat{\beta }$(95% CI)	*p*-value
*Fixed effects*						
Intercept	23.93 (− 33.35, 81.22)	0.3929	12.04 (− 0.86, 24.94)	0.0668	21.10 (4.65, 37.55)	0.0139
**Shift type**						
Day shift	0		0		0	
Night shift	− 16.61 (− 27.87, − 5.35)	0.0058	− 11.82 (− 18.35, − 5.29)	0.0006	− 6.27 (− 14.28, 1.74)	0.1200
**Study day**						
Day 1	0		0		0	
Day 2	− 7.47 (− 14.79, − 0.15)	0.0459	0.26 (− 3.78, 4.30)	0.8987	1.47 (− 2.68, 5.62)	0.4713
* Random effects*	*Variance component (SE)*		*Variance component (SE)*		*Variance component (SE)*	
Shift type	93.62 (52.80)		94.55 (29.25)		82.05 (30.66)	
Residual	116.58 (38.27)		84.27 (18.62)		42.52 (13.43)	
**C1: at waking up**^**a**^	exp ($\widehat{\beta }$)(95% CI)	*p*-value	exp ($\widehat{\beta }$)(95% CI)	*p*-value	exp ($\widehat{\beta }$)(95% CI)	*p*-value
*Fixed effects*						
Intercept	9.06 (0.80, 102.5)	0.0724	14.31 (7.80, 26.23)	< 0.0001	19.02 (8.90, 40.66)	< 0.0001
**Shift type**						
Day shift	1		1		1	
Night shift	1.41 (0.87, 2.29)	0.1506	1.01 (0.75, 1.38)	0.9268	1.23 (0.85, 1.77)	0.2652
**Study day**						
Day 1	1		1		1	
Day 2	1.63 (1.10, 2.42)	0.0169	1.23 (1.01, 1.49)	0.0373	1.05 (0.88, 1.26)	0.5865
* Random effects*	*Variance component (SE)*		*Variance component (SE)*		*Variance component (SE)*	
Shift type	0.0966 (0.11)		0.2072 (0.07)		0.1870 (0.07)	
Residual	0.3471 (0.11)		0.1972 (0.04)		0.0772 (0.03)	
**C2: at waking up + 30 min**^**a**^	exp ($\widehat{\beta }$)(95% CI)	*p*-value	exp ($\widehat{\beta }$)(95% CI)	*p*-value	exp ($\widehat{\beta }$)(95% CI)	*p*-value
*Fixed effects*						
Intercept	15.63 (1.87, 130.9)	0.0139	28.80 (16.89, 49.10)	< 0.0001	30.34 (13.67, 67.34)	< 0.0001
**Shift type**						
Day shift	1		1		1	
Night shift	0.73 (0.48, 1.11)	0.1342	0.59 (0.45, 0.78)	0.0003	0.76 (0.52, 1.11)	0.1447
**Study day**						
Day 1	1		1		1	
Day 2	1.09 (0.85, 1.40)	0.4592	1.19 (0.99, 1.43)	0.0661	1.02 (0.85, 1.24)	0.792
* Random effects*	*Variance component (SE)*		*Variance component (SE)*		*Variance component (SE)*	
Shift type	0.1443 (0.07)		0.1369 (0.06)		0.1852 (0.07)	
Residual	0.1313 (0.04)		0.1818 (0.04)		0.0845 (0.03)	

Data shown for shift workers (N = 66) on day and night shifts (n = 186 study days).

*CAR* cortisol awakening response; *CI* confidence interval; *SE* standard error.

^a^Adjusted for age (years).

**Figure 3:**
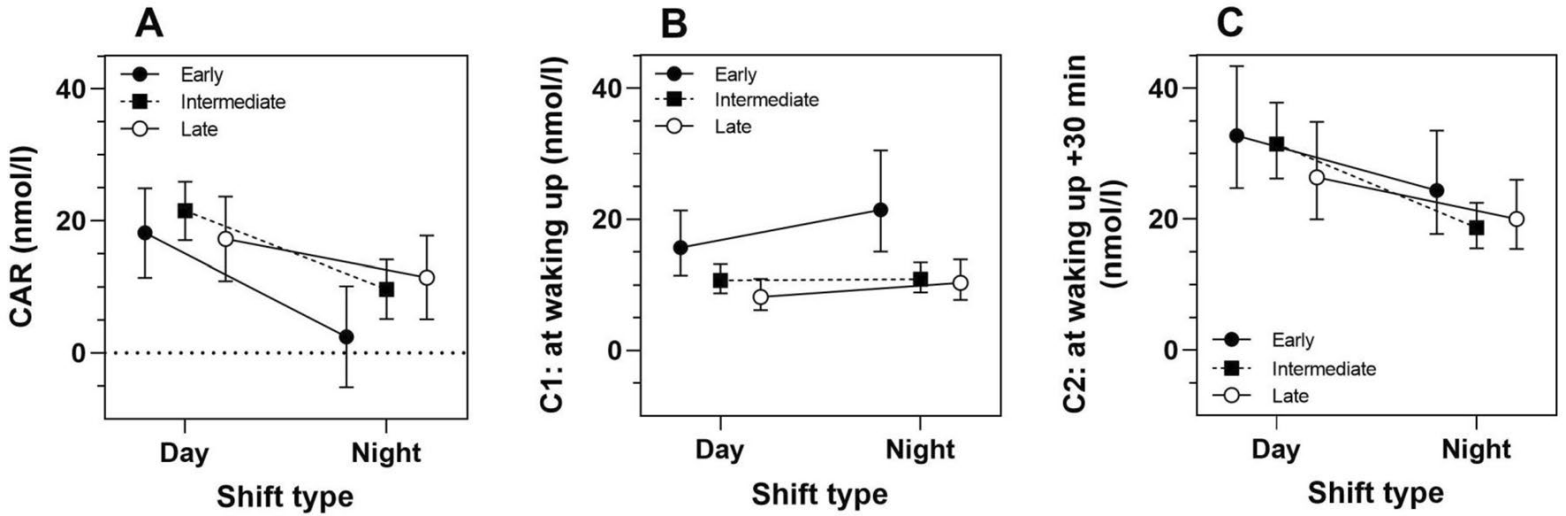
Adjusted least-squares means (LS-means) and 95% confidence intervals of salivary cortisol levels in shift workers by shift type (day, night) and chronotype group (early, intermediate, late): (**A**) CAR, (**B**) C1, and (**C**) C2. LS-means are derived from linear mixed-effects models with main terms for shift type, study day, chronotype and interaction of shift type x chronotype, adjusted for age (Supplementary Table S5). For each of the three outcomes *p*-value for the interaction shift type x chronotype was greater 0.20. The differences in CAR between day and night shifts in early chronotypes: $\widehat{\upbeta }$=-15.70, multiplicity adjusted 95% CI − 29.92, − 1.47, intermediate chronotypes: $\widehat{\upbeta }$=-11.85, 95% CI − 21.02, − 2.69, late chronotypes: $\widehat{\upbeta }$=-5.84, multiplicity adjusted 95% CI − 18.56, 6.89 (panel A). Cortisol levels in C1 were higher in early chronotypes compared to late chronotypes in both day shifts (exp $(\widehat{\upbeta })$=1.91, multiplicity adjusted 95% CI 1.01, 3.59) and night shifts (exp $(\widehat{\upbeta })$=2.07, multiplicity adjusted 95% CI 1.04, 4.12) (panel B).

Figure 3 shows LS-means derived from the LMM’s with the interaction of shift type by chronotype and main terms for shift type (night/day) and chronotype (Supplementary Table S5). There are only slightly different results between the subgroup analysis and the LMM’s with the interaction of shift type by chronotype. The differences in CAR between day and night shifts were most pronounced in early chronotypes and least pronounced in late chronotypes. There was no effect modification by chronotype on the associations between night shift and any of the three cortisol measures. For each of the three measures, the *p*-value for the interaction shift type by chronotype was greater 0.20 (Supplementary Table S5). Post-hoc analyses of LS-mean differences between chronotypes within each shift, showed that C1 levels were higher in early compared to late chronotypes (day shifts: exp $(\widehat{\beta })$=1.91, multiplicity adjusted 95% CI 1.01, 3.59; and night shifts: exp $(\widehat{\beta })$=2.07, multiplicity adjusted 95% CI 1.04, 4.12) (Fig. 3B).

## Discussion

In this study of female hospital employees, we found differences in cortisol after waking up from night sleep compared to waking up from day sleep (i.e. after night-shifts) during the first two days of their shift schedule. In shift workers, we found CAR to be significantly lower after day sleep opposed to CAR after night sleep. Shift workers after night sleep showed lower cortisol levels at C1 compared to non-shift workers after night sleep. A negative association between night shift and CAR was observed in all chronotype groups and was most pronounced in early chronotypes. Chronotype did not modify the association between night shift and neither of the cortisol measures.

Cortisol at C1 after night sleep was lower in shift workers than C1 after night sleep in non-shift workers. This difference might result from different sleep and sampling times. For day shifts, bed and wake-up times varied between shift and non-shift workers. Shift workers started their day shifts earlier, and, on average, woke up 42 min earlier than non-shift workers. This could serve as an explanation for lower cortisol levels at C1 when captured at an earlier time (phase) during its natural rising portion. This finding indicates that shift workers seem to have collected their saliva samples during a period of reduced secretory activity. This means that they took their saliva samples at a circadian phase when their bodies were (in part) biologically asleep.

The influence of time of waking up on morning cortisol was previously shown^17,29^. Williams et al.^30^ found that earlier wake-up times before a day shift were associated with lower cortisol levels at C1 and pronounced CARs among both men and women. This observation was later corroborated by Bracci et al.^31^ who observed lower morning cortisol in shift-working nurses compared to day-working nurses. In contrast, we did not find differences in CAR between day shifts.

In our study, the lower CAR in shift workers after day sleep was a consequence of lower cortisol at C2. We calculated CAR as the relative difference between cortisol at C2 and C1, but C1 levels differed only marginally between day and night shifts. The negative association between night shifts and CAR was pronounced in early chronotypes. While C2 after day sleep tended to be lower in all chronotype groups, early types showed the lowest CAR after day sleep due to increased cortisol at C1, leading to negative CARs.

Others found that early compared to late chronotypes, irrespective of their sex, showed higher cortisol in the first hour after waking up from night sleep^25,26^. In our study, chronotype-specific differences in cortisol after waking up were only observed at C1, but not at C2. This finding was independent of shift type, since we observed this difference at C1 after both night sleep and day sleep. It can be assumed that chronotype-specific differences disappear when samples are taken at comparable circadian phases. Therefore, the interpretation of physiological and behavioral differences between shift and non-shift workers can easily be confounded when circadian phase and/or chronotype is not considered. Aberrant cortisol levels were shown in populations with physiological (e.g. cardiovascular disease, obesity, autoimmune disorders, allergies) and psychological (e.g. chronic stress, fatigue, burnout, exhaustion) conditions^11–13^. The negative health outcomes that are ascribed to shift-work populations are also suspected to result from deviations in the circadian profiles of hormones and neuronal signals^1,4,32,33^. But, cortisol levels vary with time of day, which is a result of the underlying circadian component promoting high levels in the morning and a progressive decline across the day^8^. That we confirmed the expected differences in CAR between morning samples (collected after night sleep) and afternoon samples (collected after day sleep)^15–20^ indicates, therefore, that the circadian cortisol rhythm in our study participants was not shifted in phase^18,21–23^.

Strength of our study is the inter- and intraindividual comparison of cortisol levels after day and night sleep in shift and non-shift workers from the same workplace. Participants collected two saliva samples per day on two working days with day shifts and two samples per day on each two days with night shifts. Overall, participants’ adherence to the study protocol was good. Another strength is the assessment of sleep parameters using validated objective (SOMNOwatch) and subjective (MCTQ_shift_) techniques. The MCTQ_shift_ that we used is currently the only validated questionnaire to assess chronotype in shift-work populations. The MCTQ_shift_ allows to calculate chronotype as a time point. The most commonly used metric is the Morningness-Eveningness Questionnaire^25–27,34^ which gives a score based on preferred times of sleep and daytime activities. Chronotype assessment based on time, opposed to a score, is ideally suited for analyses that compare physiological markers with respect to time. The heterogeneity in the methods used to determine chronotype^35^, complicate the comparison between studies, if different chronotype metrices were applied.

A limitation of our study is the calculation of CAR from only two saliva samples, as later pointed out by Stalder et al.^10^. Further limitations are the self-reported sampling times of saliva, and a possible selection bias due to exclusion of mistimed samples. An assessment of the cortisol awakening response requires exact sampling within minutes after sleep offset. Sleep timing in shift workers can be fragmented, resulting in, for example, multiple time points of awakening or premature waking up followed by a duration of wakefulness before getting up. Such mismatch between time of waking up and saliva sampling led us to exclude a number of participants from the final analysis (Fig. 1). To reduce the potential of selection bias, we excluded samples that were taken more than 15 min after awakening (C1), which reduced the sample size by 19.8%. Post-hoc analyses including the previously excluded study days with mistimed saliva sampling did not change the direction of the findings (data not shown). Post-hoc analyses using a stricter accuracy margin for saliva sampling (± 5 min) did not change the overall direction of the findings. Only in early chronotypes the negative association between night shift and CAR was diminished (data not shown). For some of the stratified analyses we had limited power, due to the small number of study days. Night workers must sleep during the day which—due to the endogenous circadian cortisol rhythm—leads to lower cortisol after waking up in the afternoon. That mismatch between waking up and cortisol can confound the analysis, because of potential, but undetected phase shifts of the circadian clock. To avoid such confounding our participants had work-free days and no night shifts prior to the study start. More sophisticated analyses to better understand how shift work impacts on the endogenous circadian cortisol rhythm would include at least hourly saliva samples on many more workdays and also work-free days, which in its entirety is virtually impossible in a field study. Finally, we did not assess job demand in our participants and, hence, cannot fully exclude respective impact on our study findings.

In summary, early awakening after night sleep in shift workers, compared to non-shift workers, was associated with lower cortisol levels at C1. Within the shift work group, there were only marginal differences at C1 between day and night shifts, suggesting that the saliva samples were taken at a circadian phase of naturally reduced secretion. Our findings suggest a mismatch between time of waking up (both after night and day sleep) and peak of cortisol that occurs around the time of awakening. The shift-work burden at the level of the individual and society can be severe and costly^36^. For example, deficits in health and cognitive function in shift-working hospital employees pose a significant risk to patients in terms of increased medication error rates^5,37^. Being physically and mentally fit for duty is particularly important in occupations which are critical to the safety of people (e.g., pilots, police officers, fire workers, nuclear power plant operators, etc.). Hence, it is essential to better understand the adaptation processes of the circadian system in different shift work environments to help design evidence-based recommendations for healthier and safer rosters^38–40^. Future studies could help to elucidate to what extent performance and health impairments can be reduced by taking into consideration the concepts and findings presented in the current paper. Of particular importance, in our eyes, is research helping to identify reasons why chronotype-specific differences in cortisol are found in some populations but not in others.

## Methods

### Study population and design

Female hospital employees, aged 25–65 years and working either day and night shifts or day shifts only, were recruited between September 2012 and May 2015 at the University Hospital Bergmannsheil in Bochum, Germany (75 shift workers, 25 non-shift workers). The shiftwork schedule was irregular including between three to five night-shifts per month. Non-shift workers worked regular day-shifts for at least two years prior study start^41^. The study protocol included interviews and sleep apnea screenings prior to the field phases with detailed assessments of biological parameters during night and day shift periods. Exclusion criteria were current pregnancy, breast feeding in the last six months, ovarian stimulation, and a diagnosis of cancer. The study was approved by the Ruhr University Bochum Research Ethics Committee (No. 4450–12), and all participants gave written informed consent prior study start. All study protocols and methods were performed in accordance with the standards set by the Declaration of Helsinki.

Shift workers started into the follow-up on a day or night shift, whichever occurred first after recruitment into the study. Shift workers were studied on two consecutive day shifts and three consecutive night shifts. The study design included a four-week intermission between day and night shifts. The study design also included that participants had not worked in night shifts at least three days before each study period. Day shifts started between 06:00 and 07:00 h, with a duration of 8 h. Night shifts started between 21:00 and 22:00 h and lasted 9 h.

Non-shift workers were studied on two consecutive working days. Day shifts in this study group lasted 8 h and started as follows: 6:00–6:59 h (n = 7 participants), 7:00–7:59 h (n = 14), 8:00–8:59 h (n = 4).

### Saliva sampling

Saliva collection was performed using salivettes (Sarsted, Nuembrecht, Germany). Participants were asked to provide two saliva samples on two days of each day and night shift period. Beginning on the first study day and for the following study day, participants carried out saliva sampling themselves at waking up (C1) and 30 min after waking up (C2) after night sleep preceding day shifts and after day sleep following night-shift work. C1 and C2 samples were not collected after day sleep following the third night shift. Participants were asked to refrain from brushing their teeth and eating for at least 30 min prior to each sampling. Saliva sampling times were documented on corresponding saliva tubes and in a log book. Samples were cooled at participants’ homes and collected by a study nurse at the end of each shift. Samples were subsequently aliquoted and stored at − 80 °C. Cortisol levels were determined employing a commercially available chemiluminescence assay (IBL-Hamburg, Hamburg, Germany). Saliva samples were processed based on recommendations^10,42^.

### Polysomnography

Sleep was recorded at participants’ homes by applying the SOMNOwatch™ plus the Rechtschaffen and Kales sensor module (SOMNOmedics GmbH, Randersacker, Germany). The SOMNOwatch has been validated against standard polysomnographic diagnostics applied in sleep laboratories^43,44^. The Rechtschaffen and Kales sensor module provides seven electrodes: three for electroencephalography (EEG), two for electrooculography (EOG) and two for chin electromyography (EMG). Our participants were asked to wear the SOMNOwatch during sleeping hours across three consecutive study days with night shifts and two consecutive study days with day shifts. The SOMNOwatch was fitted to the thorax and provided triaxial accelerometer, ambient light and body position. Sleep onset/offset detection (for the CAR analysis) was performed by a trained sleep technologist at the sleep laboratory of the Bergmannsheil Hospital through visual inspection of the recorded sleep data in 30-s epochs^45^. The overall quality of recorded sleep data was rated as good, sufficient, or insufficient. The following three parameters were considered: bedtime, time of waking up (sleep offset), and total sleep time (sleep period minus duration of intra-sleep wake periods).

### Obstructive sleep apnea screening

Home sleep apnea tests (Easy-Screen Pro, Löwenstein Medical, Bad Ems, Germany) were performed prior to the field phases. Episodes of hypopnea and apnea in the Easy-Screen recordings were quantified by a sleep technologist of the Bergmannsheil sleep laboratory. For each participant a respiratory disturbance index (RDI) was calculated. Obstructive sleep apnea (OSA) was diagnosed as mild (RDI 5- ≤ 15), moderate (RDI ≥ 15- < 35), or severe (RDI ≥ 35).

### Questionnaire and diary data

Participants reported sociodemographic and lifestyle characteristics (age, sex, smoking status, level of education, etc.), a detailed shift-work history, health disorders, and medication in a face-to-face interview, at which also anthropometric measurements were performed. Participants completed the Munich ChronoType Questionnaire for shift-work populations (MCTQ_shift_)^28^. The MCTQ_shift_, just like the general version of the MCTQ, was designed to estimate chronotype based on the midpoint of sleep on work-free days (and in this case, on days after day shifts), corrected for sleep debt on workdays. Mid-sleep was estimated as described by Rotter et al.^46^ and was categorized using the 25th percentile (3:11 h) and 75th percentile (4:47 h) of the whole study population as cutoffs for early, intermediate and late chronotypes. Participants also completed a diary to collect information on coffee, alcohol, and medication intake for each hour of the study day.

### Outcomes

Individual CAR, as the primary outcome, was calculated as the difference between cortisol levels measured at C2 and C1. Secondary outcomes, i.e. cortisol levels at C1 and C2, were log-transformed.

### Covariates

We considered potential covariates on the basis of prior knowledge of potential influence on CAR^10^. As factors related to saliva sampling we selected: season (spring, summer, fall, winter), coffee in the first hour after waking up (yes/no), prior consumption of sleep medication or alcohol (yes/no), time of waking up and sleep duration (hours). As potentially relevant demographic or lifestyle factors we considered: age (years), cigarette smoking (never, former, current), body-mass index (normal weight < 25 kg/m^2^; overweight 25–29.9 kg/m^2^; obesity ≥ 30 kg/m^2^), obstructive sleep apnea (none, mild, moderate), and menopausal status (premenopausal, postmenopausal, surgical/other amenorrhea).

### Statistical analyses

Each of the three cortisol measures (CAR, C1, and C2) were based on repeated measures across two consecutive study days with day shift only (non-shift workers group). In the shift-workers data are based on repeated measures across two consecutive study days with day and two consecutive study days with night shift. C1 and C2 samples were collected only on the first- and second-night shift. The linear mixed-effects models (LMM) with CAR and log-transformed cortisol levels at C1 and C2 as outcomes were fitted as normally distributed with the identity link. Results of LMMs for log(C1) or log(C2) were reported as back-transformed effect estimates (exp $(\widehat{\beta })$) and 95% confidence intervals (CI). Minimally sufficient adjustment sets were identified a priori by use of directed acyclic graphs using the DAGitty software^47^. LMMs were fitted using the restricted maximum likelihood estimate of the variance components^48^ and the approximation of the degrees of freedom by Kenward-Roger method^49^. First, we examined the effect of night shift (i.e. waking up from day sleep) on each of the three outcomes in shift workers. We fitted LMM with fixed-effects for shift type, study day, and with random slope for shift type. The variance–covariance was modelled as variance compound. The minimally sufficient adjustment set for the association of shift work with awakening cortisol measures included age and chronotype (Supplementary Fig. S1). Second, we assessed the effect of study group (shift workers compared to non-shift workers) on the three awakening cortisol outcomes in day shifts. We fitted LMMs with a compound symmetry residual variance–covariance matrix. We assessed whether shift worker chronotype was a modifier for the effect of night shift on CAR, log(C1) and log(C2) in the stratified analyses and also in the LMM’s with shift type, study day, chronotype and an interaction term of shift type by chronotype. Adjusted least-squares means (LS-means) with 95% confidence intervals of awakening cortisol measures were calculated. Post-fitted comparisons between LS-means were adjusted for multiplicity by simulation^50^. The following sensitivity and stratified analyses were performed: excluding study days with self-reported use of sleep-aid or alcohol consumption, excluding study days with negative CARs, as well as stratifying for season and BMI (normal, overweight/obesity). Statistical analyses were performed in SAS version 9.4.

## Supplementary Information

Supplementary Information.
