# Supplementary material for: Night work, chronotype and cortisol at awakening in female hospital employees
Source: Sci Rep. 2022 Apr 20;12:6525. doi: 10.1038/s41598-022-10054-w (PMC9021274; doi:10.1038/s41598-022-10054-w)
Supplement: Supplementary file 1 — Supplementary Information. [file 41598_2022_10054_MOESM1_ESM.docx]

**Supplementary Material**

**Night work, chronotype and cortisol at awakening in female hospital employees**

Katarzyna Burek, Sylvia Rabstein, Thomas Kantermann, Céline Vetter, Markus Rotter, Rui Wang-Sattler, Martin Lehnert, Dirk Pallapies, Karl-Heinz Jöckel, Thomas Brüning, Thomas Behrens

**Table of Contents**

**Supplementary Figure S1 (A).** Directed acyclic graph illustrating the hypothesized association between shift work (exposure of interest) and **cortisol awakening response (CAR)** (outcome of interest).

**Supplementary Figure S1 (B).** Directed acyclic graph illustrating the hypothesized association between shift work (exposure of interest) and **cortisol at waking up (C1)** (outcome of interest).

**Supplementary Figure S1 (C).** Directed acyclic graph illustrating the hypothesized association between shift work (exposure of interest) and **cortisol at waking up +30 min (C2)** (outcome of interest).

**Supplementary Figure S2.** Bedtime, time of waking up, and sleep duration per study group (non-shift workers, shift workers), shift type (day, night) and chronotype group (early, intermediate, late).

**Supplementary Table S1.** Associations (effect estimates and 95% confidence intervals) of day shift with CAR, log(C1) and log(C2) stratified by BMI (normal weight, overweight/obesity).

**Supplementary Table S2.** Associations (effect estimates and 95% confidence intervals) of day shift with CAR, log(C1) and log(C2) stratified by season.

**Supplementary Table S3.** Results of the sensitivity analysis excluding study days (n=22) with negative CAR, showing associations (effect estimates and 95% confidence intervals) of night shift with CAR, log(C1) and log(C2). Shift workers on day and night shifts (n=164 study days).

**Supplementary Table S4.** Results of the sensitivity analysis excluding study days (n=22) with negative CAR. Associations (effect estimates and 95% confidence intervals) of night shift with CAR, log(C1) and log(C2) stratified by chronotype group. Shift workers on day and night shifts (n=164 study days).

**Supplementary Table S5.** Associations (effect estimates and 95% confidence intervals) of night shift with CAR, log(C1) and log(C2). Shift workers on day and night shifts (n=184 study days).

**
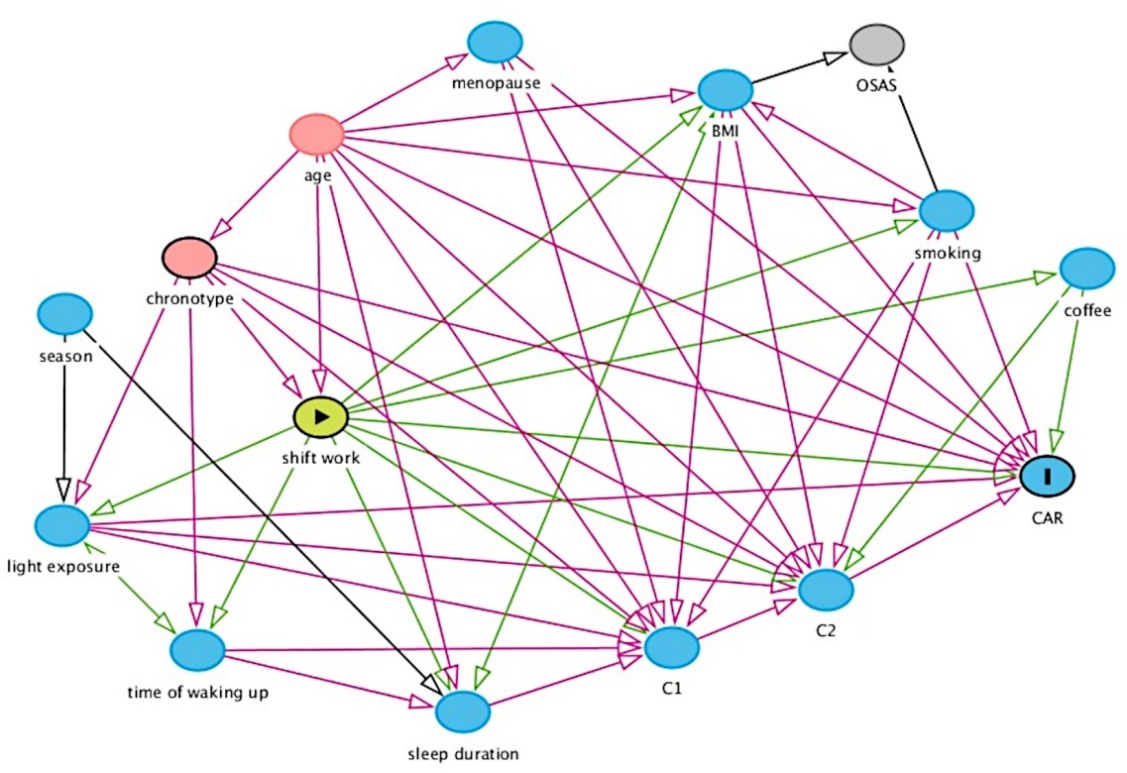
**

Legend:
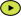
 exposure,
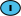
 outcome.
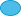
 ancestor of outcome,
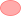
 ancestor of exposure *and* outcome,
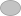
 other variable,
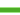
 causal path,
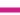
 biasing path.

**Supplementary Figure S1 (A).** Directed acyclic graph illustrating the hypothesized association between shift work (exposure of interest) and **cortisol awakening response (CAR)** (outcome of interest).

**
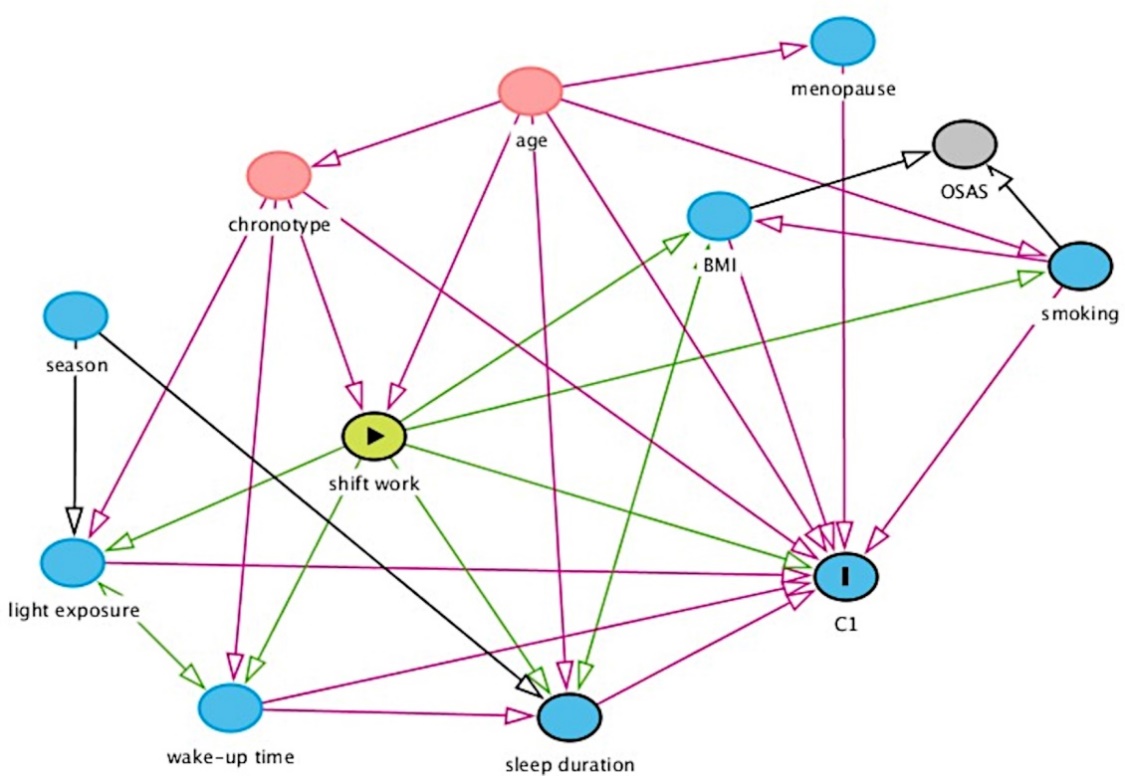
**

Legend:
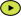
 exposure,
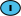
 outcome.
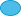
 ancestor of outcome,
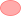
 ancestor of exposure *and* outcome,
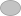
 other variable,
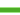
 causal path,
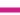
 biasing path.

**Supplementary Figure S1 (B).** Directed acyclic graph illustrating the hypothesized association between shift work (exposure of interest) and **cortisol at waking up (C1)** (outcome of interest).

**
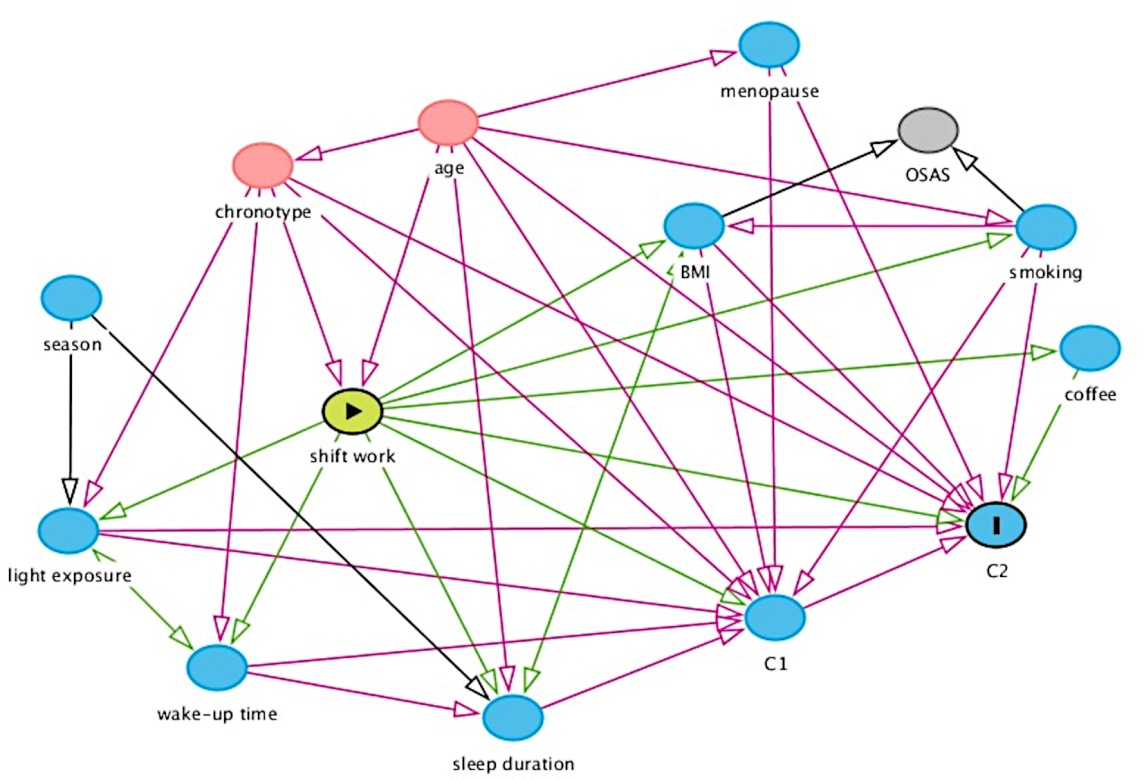
**

Legend:
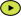
 exposure,
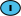
 outcome.
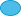
 ancestor of outcome,
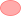
 ancestor of exposure *and* outcome,
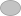
 other variable,
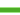
 causal path,
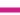
 biasing path.

**Supplementary Figure S1 (C).** Directed acyclic graph illustrating the hypothesized association between shift work (exposure of interest) and **cortisol at waking up +30 min (C2)** (outcome of interest).

**
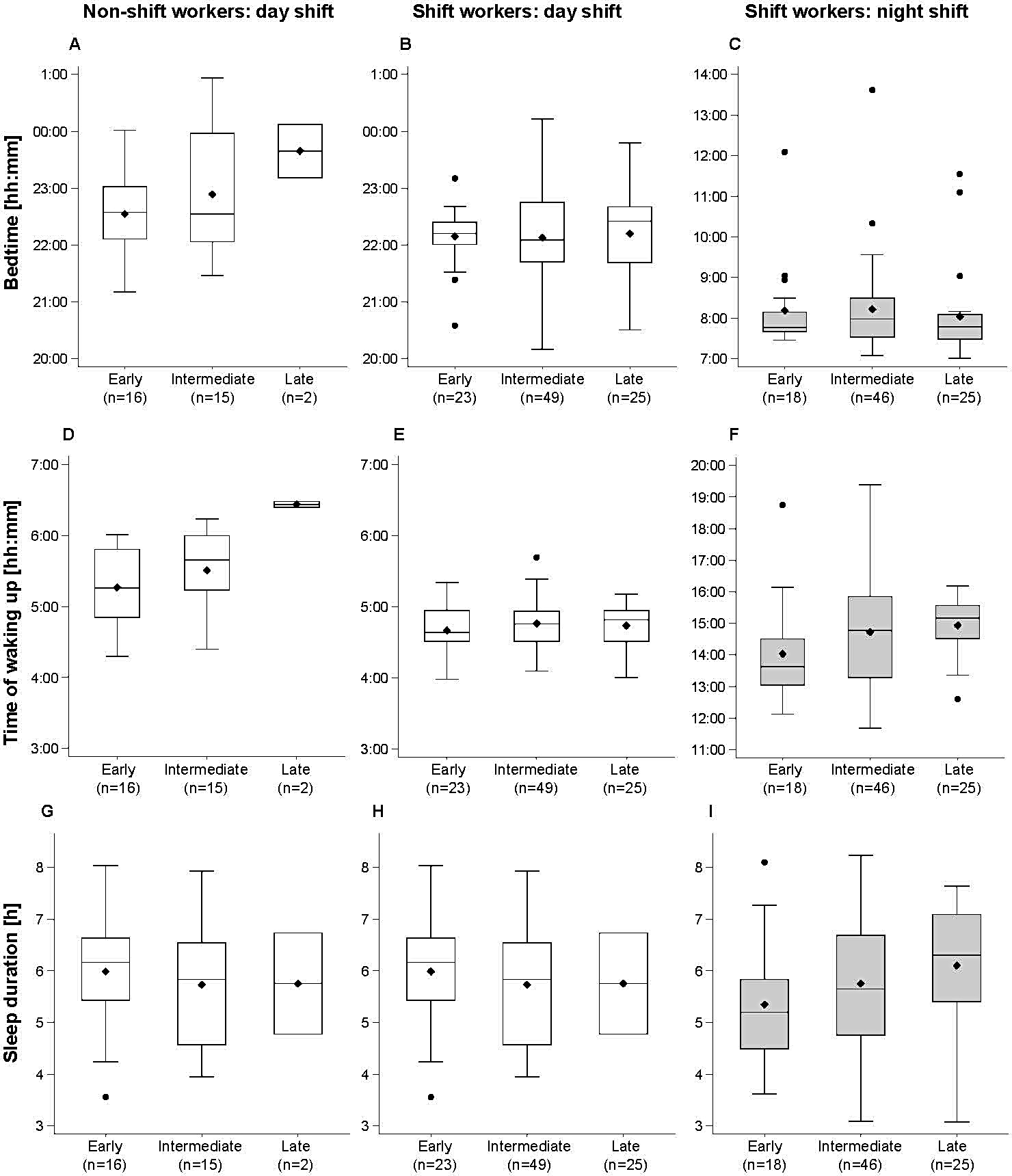
**

**Supplementary Figure S2. Bedtime, time of waking up, and sleep duration per study group (non-shift workers, shift workers), shift type (day, night) and chronotype group (early, intermediate, late).** Non-shift workers: day shift (left panels: A, D, G), shift workers: day shift (middle panels: B, E, H) and shift workers: night shift (right panels: C, F, I). No differences between the chronotype groups in sleep parameters in each of the three groups after adjustment for multiplicity. Study day’s numbers (n) per chronotype group are shown in the figure. Boxplots are Tukey boxplots with whiskers encompassing all data points within 1.5 times the interquartile range.

**Supplementary Table S1.** Associations (effect estimates and 95% confidence intervals) of day shift with CAR, salivary cortisol at waking up (C1) and salivary cortisol 30 min after waking up (C2) stratified by BMI (normal weight, overweight/obesity).

|  | Non-shift and shift workers on day shifts (n=134 study days) | | | |  | Shift workers on day and night shifts (n=186 study days) | | | |
| --- | --- | --- | --- | --- | --- | --- | --- | --- | --- |
|  | Normal weight (<25 kg/m^2^)  (n=70) | | Overweight/obesity (≥25 kg/m^2^)  (n=64) | |  | Normal weight (<25 kg/m^2^)  (n=95) | | Overweight/obesity (≥25 kg/m^2^)  (n=91) | |
| **CAR^a^** | | | | |  |  |  |  |  |
|  | $\hat{\beta}$ (95% CI) | *p-*value | $\hat{\beta}$ (95% CI) | *p-*value |  | $\hat{\beta}$ (95% CI) | *p-*value | $\hat{\beta}$ (95% CI) | *p-*value |
| *Fixed effects* |  |  |  |  |  |  |  |  |  |
| Intercept | 17.66 (-12.99, 48.31) | 0.2512 | 13.12 (-26.01, 52.25) | 0.5006 |  | 16.35 (-6.06, 38.76) | 0.1496 | 9.71 (-16.82, 36.23) | 0.4654 |
| Day shift  Non-shift workers  Shift workers | 0  3.48 (-5.95, 12.91) | 0.4603 | 0  -1.06 (-13.30, 11.18) | 0.8618 |  | n.a. | n.a. | n.a. | n.a. |
| Shift type  Day shift  Night shift | n.a. | n.a. | n.a. | n.a. |  | 0  -11.67 (-17.4, -5.92) | 0.0002 | 0  -10.12 (-17.69, -2.56) | 0.0097 |
| Study day  Day 1  Day 2 | 0  -0.66 (-2.85, 1.52) | 0.5494 | 0  -0.85 (-4.27, 2.67) | 0.6219 |  | 0  1.22 (-1.82, 4.26) | 0.4212 | 0  -3.36 (-8.14, 1.41) | 0.1633 |
| *Random effects* | Variance component (SE) | | Variance component (SE) | |  | Variance component (SE) | | Variance component (SE) | |
| Intercept | 136.84 (33.61) | | 204.01 (55.66) | |  | n.a. | | n.a. | |
| Shift type | n.a. | | n.a. | |  | 85.51 (22.62) | | 109.45 (37.88) | |
| Residual | 41.83 (5.80) | | 94.0 (13.67) | |  | 44.18 (10.25) | | 119.87 (26.04) | |
| **C1: at waking up^a^** | | | | |  |  |  |  |  |
|  | exp$(\hat{\beta})$ (95% CI) | *p-*value | exp$(\hat{\beta})$ (95% CI) | *p-*value |  | exp$(\hat{\beta})$ (95% CI) | *p-*value | exp$(\hat{\beta})$ (95% CI) | *p-*value |
| *Fixed effects* |  |  |  |  |  |  |  |  |  |
| Intercept | 117.5 (34.88, 395.72) | <0.0001 | 22.38 (5.00, 100.2) | 0.0002 |  | 68.43 (25.40, 184.4) | <0.0001 | 22.56 (7.03, 72.43) | <0.0001 |
| Day shift  Non-shift workers  Shift workers | 1  0.59 (0.41, 0.85) | 0.0063 | 1  0.52 (0.30, 0.92) | 0.0251 |  | n.a. | n.a. | n.a. | n.a. |
| Shift type  Day shift  Night shift | n.a. | n.a. | n.a. | n.a. |  | 1  1.09 (0.85, 1.40) | 0.5035 | 1  1.24 (0.89, 1.73) | 0.2044 |
| Study day  Day 1  Day 2 | 1  1.12 (0.89, 1.41) | 0.3269 | 1  1.64 (1.27, 2.12) | 0.0005 |  | 1  1.03 (0.86, 1.23) | 0.7426 | 1  1.52 (1.23, 1.87) | 0.0002 |
| *Random effects* | Variance component (SE) | | Variance component (SE) | |  | Variance component (SE) | | Variance component (SE) | |
| Intercept | 0.0676 (0.05) | | 0.2593 (0.10) | |  | n.a. | | n.a. | |
| Shift type | n.a. | | n.a. | |  | 0.1165 (0.05) | | 0.2123 (0.08) | |
| Residual | 0.2113 (0.05) | | 0.2273 (0.06) | |  | 0.1682 (0.04) | | 0.2292 (0.05) | |
| **C2: at waking up +30 min^a^** | | | | |  |  |  |  |  |
|  | exp$(\hat{\beta})$ (95% CI) | *p-*value | exp$(\hat{\beta})$ (95% CI) | *p-*value |  | exp$(\hat{\beta})$ (95% CI) | *p-*value | exp$(\hat{\beta})$ (95% CI) | *p-*value |
| *Fixed effects* |  |  |  |  |  |  |  |  |  |
| Intercept | 54.24 (18.79, 156.6) | <0.0001 | 22.90 (7.28, 72.01) | <0.0001 |  | 65.40 (22.25, 192.2) | <0.0001 | 26.98 (10.50, 69.34) | <0.0001 |
| Day shift  Non-shift workers  Shift workers | 1  0.96 (0.69, 1.33) | 0.7875 | 1  0.80 (0.57, 1.13) | 0.2074 |  | n.a. | n.a. | n.a. | n.a. |
| Shift type  Day shift  Night shift | n.a. | n.a. | n.a. | n.a. |  | 1  0.63 (0.48, 0.83) | 0.0016 | 1  0.71 (0.55, 0.93) | 0.0138 |
| Study day  Day 1  Day 2 | 1  1.11 (0.97, 1.26) | 0.1140 | 1  1.15 (0.97, 1.37) | 0.0979 |  | 1  1.16 (1.01, 1.33) | 0.0387 | 1  1.09 (0.90, 1.31) | 0.3723 |
| *Random effects* | Variance component (SE) | | Variance component (SE) | |  | Variance component (SE) | | Variance component (SE) | |
| Intercept | 0.1385 (0.04) | | 0.1156 (0.05) | |  | n.a. | | n.a. | |
| Shift type | n.a. | | n.a. | |  | 0.2068 (0.06) | | 0.1083 (0.05) | |
| Residual | 0.0768 (0.02) | | 0.1217 (0.03) | |  | 0.0884 (0.02) | | 0.1834 (0.04) | |

Abbreviations: CAR, cortisol awakening response; CI, confidence interval; SE, standard error.

^a^ Adjusted for age (years), and chronotype (MCTQ_shift_, clock times).

**Supplementary Table S2.** Associations (effect estimates and 95% confidence intervals) of day shift with CAR, salivary cortisol at waking up (C1) and salivary cortisol 30 min after waking up (C2) stratified by season.

|  | Non-shift and shift workers on day shifts (n=134 study days) | | | |  | Shift workers on day and night shifts (n=186 study days) | | | |
| --- | --- | --- | --- | --- | --- | --- | --- | --- | --- |
|  | Spring/summer (n=64) | | Fall/winter (n=70) | |  | Spring/summer (n=99) | | Fall/winter (n=87) | |
| **CAR^a^** | | | | |  |  |  |  |  |
|  | $\hat{\beta}$ (95% CI) | *p-*value | $\hat{\beta}$ (95% CI) | *p-*value |  | $\hat{\beta}$ (95% CI) | *p-*value | $\hat{\beta}$ (95% CI) | *p-*value |
| *Fixed effects* |  |  |  |  |  |  |  |  |  |
| Intercept | 6.35 (-30.95, 43.65) | 0.7305 | 30.09 (-8.82, 69.0) | 0.1255 |  | -0.21 (-21.18, 20.76) | 0.9841 | 29.90 (1.96, 57.8) | 0.0364 |
| Day shift  Non-shift workers  Shift workers | 0  1.07 (-9.85, 11.99) | 0.8429 | 0  5.11 (-8.08, 18.30) | 0.4371 |  | n.a. | n.a. | n.a | n.a. |
| Shift type  Day shift  Night shift | n.a. | n.a. | n.a. | n.a. |  | 0  -7.78 (-13.63, -1.92) | 0.0101 | 0  -13.97 (-21.40, -6.55) | 0.0004 |
| Study day  Day 1  Day 2 | 0  -0.57 (-4.59, 3.44) | 0.7719 | 0  -2.87 (-9.07, 3.33) | 0.3542 |  | 0  -1.23 (-5.29, 2.84) | 0.5475 | 0  -1.26 (-5.38, 2.85) | 0.5387 |
| *Random effects* | Variance component (SE) | | Variance component (SE) | |  | Variance component (SE) | | Variance component (SE) | |
| Intercept | 110.36 (37.08) | | 147.43 (55.86) | |  | n.a. | | n.a. | |
| Shift type | n.a. | | n.a. | |  | 62.25 (24.27) | | 113.64 (33.56) | |
| Residual | 49.89 (14.25) | | 145.56 (36.62) | |  | 92.83 (19.70) | | 78.50 (18.10) | |
| **C1: at waking up^a^** | | | | |  |  |  |  |  |
|  | exp$(\hat{\beta})$ (95% CI) | *p-*value | exp$(\hat{\beta})$ (95% CI) | *p-*value |  | exp$(\hat{\beta})$ (95% CI) | *p-*value | exp$(\hat{\beta})$ (95% CI) | *p-*value |
| *Fixed effects* |  |  |  |  |  |  |  |  |  |
| Intercept | 12.97 (2.17, 77.43) | 0.0064 | 70.06 (21.91, 224.1) | <0.0001 |  | 25.46 (9.21, 70.40) | <0.0001 | 35.95 (10.11, 127.8) | <0.0001 |
| Day shift  Non-shift workers  Shift workers | 1  0.75 (0.45, 1.28) | 0.2832 | 1  0.53 (0.34, 0.85) | 0.0088 |  | n.a. | n.a. | n.a. | n.a. |
| Shift type  Day shift  Night shift | n.a. | n.a. | n.a. | n.a. |  | 1  1.14 (0.86, 1.51) | 0.3480 | 1  1.11 (0.80, 1.56) | 0.5171 |
| Study day  Day 1  Day 2 | 1  1.20 (0.98, 1.48) | 0.0756 | 1  1.47 (1.11, 1.93) | 0.0080 |  | 1  1.24 (1.03, 1.49) | 0.0241 | 1  1.26 (1.00, 1.58) | 0.0491 |
| *Random effects* | Variance component (SE) | | Variance component (SE) | |  | Variance component (SE) | | Variance component (SE) | |
| Intercept | 0.2461 (0.08) | | 0.1057 (0.08) | |  | n.a. | | n.a. | |
| Shift type | n.a. | | n.a. | |  | 0.1646 (0.06) | | 0.1823 (0.08) | |
| Residual | 0.1339 (0.04) | | 0.3101 (0.08) | |  | 0.1893 (0.04) | | 0.2457 (0.06) | |
| **C2: at waking up +30 min^a^** | | | | |  |  |  |  |  |
|  | exp$(\hat{\beta})$ (95% CI) | *p-*value | exp$(\hat{\beta})$ (95% CI) | *p-*value |  | exp$(\hat{\beta})$ (95% CI) | *p-*value | exp$(\hat{\beta})$ (95% CI) | *p-*value |
| *Fixed effects* |  |  |  | |  |  |  |  |  |
| Intercept | 24.95 (7.02, 88.66) | <0.0001 | 62.89 (19.20, 206.0) | <0.0001 |  | 25.61 (11.14, 58.90) | <0.0001 | 55.96 (16.77, 186.8) | <0.0001 |
| Day shift  Non-shift workers  Shift workers | 1  0.89 (0.62, 1.30) | 0.5421 | 1  0.92 (0.63, 1.36) | 0.6713 |  | n.a. | n.a. | n.a. | n.a. |
| Shift type  Day shift  Night shift | n.a. | n.a. | n.a. | n.a. |  | 1  0.80 (0.63, 1.00) | 0.0544 | 1  0.53 (0.39, 0.73) | 0.0002 |
| Study day  Day 1  Day 2 | 1  1.05 (0.93, 1.19) | 0.3968 | 1  1.08 (0.91, 1.29) | 0.3527 |  | 1  1.12 (0.96, 1.31) | 0.1477 | 1  1.11 (0.92, 1.33) | 0.2579 |
| *Random effects* | Variance component (SE) | | Variance component (SE) | |  | Variance component (SE) | | Variance component (SE) | |
| Intercept | 0.1351 (0.04) | | 0.1330 (0.05) | |  | n.a. | | n.a. | |
| Shift type | n.a. | | n.a. | |  | 0.1068 (0.04) | | 0.1880 (0.06) | |
| Residual | 0.0455 (0.01) | | 0.1080 (0.03) | |  | 0.1319 (0.03) | | 0.1522 (0.04) | |

Abbreviations: CAR, cortisol awakening response; CI, confidence interval; SE, standard error.

^a^Adjusted for age (years), and chronotype (MCTQ_shift_, clock times).

**Supplementary Table S3.** Results of the sensitivity analysis excluding study days (n=22) with negative CAR, showing associations (effect estimates and 95% confidence intervals) of night shift with CAR, salivary cortisol at waking up (C1) and salivary cortisol 30 min after waking up (C2). Shift workers on day and night shifts (n=164 study days).

|  | CAR^a^ | |  | C1: at waking up^a^ | |  | C2: at waking up +30 min^a^ | |
| --- | --- | --- | --- | --- | --- | --- | --- | --- |
|  | $\hat{\beta}$ (95% CI) | *p-*value |  | exp$(\hat{\beta})$ (95% CI) | *p-*value |  | exp$(\hat{\beta})$ (95% CI) | *p-*value |
| *Fixed effects* |  |  |  |  |  |  |  |  |
| Intercept | 14.82 (-1.36, 31.01) | 0.0722 |  | 19.92 (9.06, 43.81) | <0.0001 |  | 25.57 (12.66, 51.65) | <0.0001 |
| Shift type  Day shift  Night shift | 0  -8.32 (-12.70, -3.94) | 0.0003 |  | 1  1.05 (0.85, 1.30) | 0.6623 |  | 1  0.75 (0.63, 0.88) | 0.0006 |
| Study day  Day 1  Day 2 | 0  -0.36 (-3.09, 2.36) | 0.7911 |  | 1  1.24 (1.08, 1.43) | 0.0034 |  | 1  1.11 (0.99, 1.23) | 0.0684 |
| *Random effects* | Variance component (SE) | |  | Variance component (SE) | |  | Variance component (SE) | |
| Shift type  Residual | 78.26 (17.43)  64.75 (11.04) | |  | 0.1707 (0.04)  0.1827 (0.03) | |  | 0.0995 (0.03)  0.1038 (0.02) | |

Abbreviations: CAR, cortisol awakening response; CI, confidence interval; SE, standard error.

^a^Adjusted for age, and chronotype (MCTQ_shift_, clock times).

**Supplementary Table S4.** Results of the sensitivity analysis excluding study days (n=22) with negative CAR. Associations (effect estimates and 95% confidence intervals) of night shift with CAR, salivary cortisol at waking up (C1) and salivary cortisol 30 min after waking up (C2) stratified by chronotype group. Shift workers on day and night shifts (n=164 study days).

|  | Early chronotype (n=34) | | Intermediate chronotype (n=84) | | Late chronotype (n=44) | |
| --- | --- | --- | --- | --- | --- | --- |
| **CAR^a^** | | | | | | |
|  | $\hat{\beta}$ (95% CI) | *p-*value | $\hat{\beta}$ (95% CI) | *p-*value | $\hat{\beta}$ (95% CI) | *p-*value |
| *Fixed effects* |  |  |  |  |  |  |
| Intercept | 20.86 (-34.61, 76.34) | 0.4414 | 13.42 (0.70, 26.13) | 0.0391 | 19.55 (4.07, 35.03) | 0.0155 |
| Shift type  Day shift  Night shift | 0  -10.29 (-21.44, 0.85) | 0.0685 | 0  -9.28 (-15.70, -2.85) | 0.0054 | 0  -5.67 (-13.20, 1.87) | 0.1338 |
| Study day  Day 1  Day 2 | 0  -4.07 (-10.22, 2.09) | 0.1798 | 0  -0.50 (-4.72, 3.73) | 0.8137 | 0  2.29 (-2.22, 6.79) | 0.3024 |
| *Random effects* | Variance component (SE) | | Variance component (SE) | | Variance component (SE) | |
| Shift type | 108.14 (46.34) | | 81.12 (26.85) | | 60.94 (26.86) | |
| Residual | 53.61 (21.16) | | 78.91 (18.62) | | 43.33 (14.83) | |
| **C1: at waking up^a^** | | | | | | |
|  | exp$(\hat{\beta})$ (95% CI) | *p-*value | exp$(\hat{\beta})$ (95% CI) | *p-*value | exp$(\hat{\beta})$ (95% CI) | *p-*value |
| *Fixed effects* |  |  |  |  |  |  |
| Intercept | 10.21 (0.69, 150.3) | 0.0857 | 12.34 (6.48, 23.48) | <0.0001 | 13.47 (6.80, 26.70) | <0.0001 |
| Shift type  Day shift  Night shift | 1  1.21 (0.70, 2.10) | 0.4733 | 1  0.96 (0.70, 1.34) | 0.8246 | 1  1.11 (0.80, 1.55) | 0.5164 |
| Study day  Day 1  Day 2 | 1  1.53 (1.01, 2.32) | 0.0433 | 1  1.28 (1.04, 1.59) | 0.0229 | 1  1.03 (0.85, 1.26) | 0.7266 |
| *Random effects* | Variance component (SE) | | Variance component (SE) | | Variance component (SE) | |
| Shift type | 0.1623 (0.13) | | 0.2077 (0.07) | | 0.1184 (0.06) | |
| Residual | 0.2537 (0.11) | | 0.2019 (0.05) | | 0.0823 (0.03) | |
| **C2: at waking up +30 min^a^** | | | | | | |
|  | exp$(\hat{\beta})$ (95% CI) | *p-*value | exp$(\hat{\beta})$ (95% CI) | *p-*value | exp$(\hat{\beta})$ (95% CI) | *p-*value |
| *Fixed effects* |  |  |  |  |  | |
| Intercept | 24.74 (2.74, 223.1) | 0.0067 | 26.27 (16.92, 40.77) | <0.0001 | 32.80 (16.05, 67.02) | <0.0001 |
| Shift type  Day shift  Night shift | 1  0.78 (0.50, 1.20) | 0.2417 | 1  0.69 (0.56, 0.87) | 0.0017 | 1  0.83 (0.58, 1.17) | 0.2716 |
| Study day  Day 1  Day 2 | 1  1.06 (0.84, 1.34) | 0.5728 | 1  1.16 (0.98, 1.38) | 0.0812 | 1  1.09 (0.91, 1.31) | 0.3072 |
| *Random effects* | Variance component (SE) | | Variance component (SE) | | Variance component (SE) | |
| Shift type | 0.1754 (0.08) | | 0.0696 (0.03) | | 0.1450 (0.06) | |
| Residual | 0.0715 (0.03) | | 0.1365 (0.03) | | 0.0660 (0.02) | |

Abbreviations: CAR, cortisol awakening response; CI, confidence interval; SE, standard error.

^a^Adjusted for age (years).

**Supplementary Table S5.** Associations (effect estimates and 95% confidence intervals) of night shift with CAR, salivary cortisol at waking up (C1) and salivary cortisol 30 min after waking up (C2). Shift workers on day and night shifts (n=186 study days).

|  | CAR^a^ | | C1: at waking up^a^ | | C2: at waking up +30 min^a^ | |
| --- | --- | --- | --- | --- | --- | --- |
|  | $\hat{\beta}$ (95% CI) | *p-*value | exp$(\hat{\beta})$ (95% CI) | *p-*value | exp$(\hat{\beta})$ (95% CI) | *p-*value |
| *Fixed effects* |  |  |  |  |  |  |
| Intercept | 17.31 (6.72, 27.89) | 0.0016 | 15.59 (9.59, 25.34) | <0.0001 | 30.16 (19.34, 47.04) | <0.0001 |
| Shift type  Day shift  Night shift | 0  -11.85 (-18.16, -5.54) | 0.0003 | 1  1.02 (0.76, 1.36) | 0.9030 | 1  0.59 (0.46, 0.77) | 0.0001 |
| Study day  Day 1  Day 2 | 0  -1.23 (-4.07, 1.62) | 0.3942 | 1  1.25 (1.09, 1.44) | 0.0017 | 1  1.12 (1.00, 1.26) | 0.0589 |
| Chronotype  Intermediate  Early  Late | 0  -3.35 (-11.50, 4.80)  -4.23 (-11.97, 3.51) | 0.4170  0.2813 | 1  1.46 (1.00, 2.12)  0.77 (0.54, 1.09) | 0.0485  0.1333 | 1  1.04 (0.74, 1.46)  0.84 (0.60, 1.17) | 0.8158  0.2939 |
| Shift type × chronotype  Day shift/intermediate  Day shift/early  Day shift/late  Night shift/intermediate  Night shift/early  Night shift/late | 0  0  0  0  -3.85 (-15.49, 7.79)  6.01 (-4.78, 16.81) | 0.5134  0.2719 | 1  1  1  1  1.35 (0.79, 2.30)  1.24 (0.76, 2.03) | 0.2714  0.3878 | 1  1  1  1  1.25 (0.77, 2.03)  1.28 (0.81, 2.01) | 0.3527  0.2846 |
| *Random effects* | Variance component (SE) | | Variance component (SE) | | Variance component (SE) | |
| Shift type  Residual | 87.35 (20.15)  85.43 (13.29) | | 0.1694 (0.04)  0.2091 (0.03) | | 0.1518 (0.04)  0.1424 (0.02) | |
| *Type III test of fixed effects* |  | |  | |  | |
| Shift type | F_1, 104.2_ = 20.63, *p*<0.0001 | | F_1, 103.6_ = 2.84, *p*=0.0952 | | F_1, 97.28_ = 12.74, *p*=0.0006 | |
| Study day | F_1, 94.9_ = 0.73, *p*=0.3942 | | F_1, 95.29_ = 10.40, *p*=0.0017 | | F_1, 87.39_ = 3.66, *p*=0.0589 | |
| Chronotype | F_2, 103.3_ = 1.41, *p*=0.2495 | | F_2, 102.6_ = 8.70, *p*=0.0003 | | F_2, 96.37_ = 0.92, *p*=0.4036 | |
| Shift type × chronotype | F_2, 104.3_ = 1.17, *p*=0.3143 | | F_2, 103.7_ = 0.76, *p*=0.4702 | | F_2, 97.34_ = 0.78, *p*=0.4626 | |

*Models for the three outcomes contain the main terms for shift work, study day, chronotype and the shift work x chronotype interaction term. The effect of shift work is described by both the main term for shift work and the shift work x chronotype interaction term. The effect of chronotype is described by both the main term for chronotype and shift work x chronotype interaction term.*

Abbreviations: CAR, cortisol awakening response; CI, confidence interval; SE, standard error.

^a^Adjusted for age (years).
